# Clinical Cases

**Published:** 1887-10

**Authors:** G. W. Sherbino

**Affiliations:** Abilene, Texas


### CLINICAL CASES.

G. W. Sherbino, M. D., Abilene, Texas.

*Otalgia Nervosa, Sanicula, Swan DMM.*—Mrs. S., æt. forty-two. Nervous temperament; dark hair and eyes; predominance of mind over body.

Overwork always brings on nervous prostration, this time affecting the teeth and head. The pain commenced in the first right upper incisor, which had been plugged with gold a year ago. Tooth feels too long and extremely sore; a dull aching, and at times pulsating; feels congested; feels as if picking it so it would bleed would relieve it (Bell.). Pain boring, extending to the bones of the nose, around the right eye into the temple and forehead. Gum-boil on the alveolar process. Aggravation from contact; from touching the tongue to the tooth; from compressing the upper lip; from chewing or eating (feels ulcerated at the root); motion.

Amelioration: From lying down, from sleeping; the pain commences again in the morning, as soon as waking, opening the eyes, and motion (Bry. alb.). As I was passing by where the patient was lying she asked me to put my hand on the back of her neck; *from the occiput down to the shoulders was a cold, clammy perspiration, as cold as if a cloth had been lain on the neck dipped in ice-water.*

I gave first Bell.$^{cm}$, one dose. Next day, no better, ℞ Bry. alb.$^{cm}$, one dose. No change. At three P. M. I obtained the cold, clammy neck, *which is one of the red strings* in this remedy.

Some will say give the DMM. I have found the 30 C, 10 M, 50 MMM, CMM, reliable, if they are indicated, and I wanted to test this remedy in this potency. She commenced to get better right away. Next morning she was well; could clinch her teeth together; soreness all gone; felt perfectly well every way, and did a large day's work for her. One dose was all she got or needed. It is foolish to give more.

*Hœmorrhoids, Nux vomica.*$^{2000}$—A lawyer, dark hair and eyes; calls himself a bilious temperament; is troubled with indigestion; tongue coated on the base; yellowish coat; poor appetite; melancholy; does not want company; desires to be alone; constipated, stool not very large; urging to evacuate the bowels in the morning; sensation after stool as though more remained (Lycopod.).

Two or three hæmorrhoidal knobs were inflamed and sore, so he could hardly walk. This attack had commenced a week before the first time he ever tried our treatment, and the reason he came was he always thought the old school commence at the wrong end to cure hæmorrhoids. He said, if you cure me you will have another convert in town. One dose of 2000th. S. L. to last a week. No return of the trouble.

*Hœmorrhoids, Nux vomica*$^{cm}$ (F.).—Mr. R., æt. fifty-five. I was called to see patient, who was lying in bed, was not able to be up. I asked him what he was suffering with, and his answer was piles; that three doctors had been attending him for five or six weeks, and begged of me not to experiment on him any more. I told him my business was to experiment on the healthy, and not on the sick. I asked him how they had got him down, and he said it seemed as though they could not torture him enough. "They squeezed the hæmorrhoids and mashed them up with instruments, and, my God! I could not tell you what they did not do. I am ten times worse off than when they commenced with me." I made an ocular inspection and found that he had piles in an aggravated form; the anus was completely filled with the piles. He had no appetite; tongue very much coated; bowels constipated; stools large and difficult; bowels only moved every two or three days, but the most aggravating symptom was he was constantly inclined to go out, but with all the straining and effort his bowels would not move (this was worse in the morning); he was nervous, could not bear to have any one come about him; irritable in the morning; worse an hour or two after meals; nothing seemed to agree with his stomach. A dose or two of Nux and in a week or two he was able to be up and around again. He was operated on for the same thing seven years ago. It has been a year and over and no relapse has occurred.

*Erysipelas, Apis m.*$^{cm}$ (F.)—Master Otto, æt. seven, while playing with children fell upon the left elbow, bruising it considerably. The next day it was quite sore and inflamed, and it kept on spreading up the arm and down the arm. Small vescicles filled with water; the inflammation was red. Swelling extended to the shoulder; down the arm to the wrist large blisters commenced forming.

He was irritable and it was hard to do anything with him. Cried a great deal; nothing pleased him; everything made him angry, then he would have a good cry. He was very thirsty for large quantities of water; urine very profuse and pale; he had to get up every half hour during the night to urinate. It was a difficult matter to get any symptoms from him ; he said it burned and stung, but that was doubtful. He wanted nothing but cold cloth, wet in cold water, put around the arm ; when it would get warm he would ask to have it put on cool again.

First day.—Gave one dose of Apis m.$^{1000}$.

Second day.—No better ; temperature, 103°; pulse, 140 ; one dose.

Third day.—Arm badly swollen from shoulder to hand.

Fourth day.—Gave one dose of CM. In one hour afterward the nurse said he broke out in a very profuse perspiration.

Fifth day.—Arm very much better, swelling decreasing very fast. Discharged cured.

Hering's *Guiding Symptoms* gives thirst so violent that she would like to drink all the time in typhoid.

Thirst in some cases, in others none. Apis, like Puls., is generally thirstless.

Urine sometimes too profuse. Great secretion of urine of a pale or straw color, depositing a reddish or brick-dust sediment; frequent urging to urinate, with copious discharge of straw-colored urine.

Frequent and excessively profuse discharge of normal urine.

